# Potential role of lipoxin in the management of COVID-19: a narrative review

**DOI:** 10.1007/s10787-022-01070-3

**Published:** 2022-09-16

**Authors:** Gaber El-Saber Batiha, Ali I. Al-Gareeb, Engy Elekhnawy, Hayder M. Al-kuraishy

**Affiliations:** 1 Department of Pharmacology and Therapeutics, Faculty of Veterinary Medicine, Damanhour University, Damanhour, 22511 AlBeheira Egypt; 2 Department of Pharmacology, Toxicology and Medicine, College of Medicine, Al-Mustansiriyah University, Baghdad, 14132 Iraq; 3 Pharmaceutical Microbiology Department, Faculty of Pharmacy, Tanta University, Tanta, 31527 Egypt

**Keywords:** Arachidonic acid, COVID-19, Inflammation, Lipoxins, SARS-CoV-2

## Abstract

Severe acute respiratory syndrome coronavirus (SARS-CoV-2) infection leads to the development of coronavirus disease 2019 (COVID-19), which causes endothelial dysfunction (ED), oxidative stress (OS), and inflammatory disorders. These changes cause hypoxia and cytokine storm with the development of cardio-pulmonary complications. Bioactive lipids and other polyunsaturated fatty acids participate in a vital role in the SARS-CoV-2 infection process. One of these mediators is the anti-inflammatory compound, lipoxin (LX). LXs are produced from arachidonic acid (AA) by collaboration between 5-lipoxygenase (5-LO) and 12–15 LO during cell interactions. Thus, our goal was to review the probable role of LXs in COVID-19 regarding the effects of LXs on the inflammatory signaling pathways that are linked with COVID-19 pathogenesis and complications.

## Background

Severe acute respiratory syndrome coronavirus (SARS-CoV-2) was primarily documented as the probable cause of acute respiratory infection in Wuhan, Hubei province of China in late December 2019 (Al-Kuraishy et al. 2021). The World Health Organization (WHO) designated this infection as coronavirus disease 2019 (COVID-19) (Attallah et al. 2021). SARS-CoV-2 utilizes angiotensin-converting enzyme type 2 (ACE2) as an entry point (Al-Kuraishy et al. 2020). Interaction of SARS-CoV-2 with ACE2 provokes a series of inflammatory changes causing cell injury and hyperinflammation (Al-Kuraishy et al. 2021). ACE2 is broadly distributed in a diverse cellular system including enterocytes, cardiomyocytes, pulmonary alveolar cells, neurons, and testes (Al-Kuraishy et al. 2021). The clinical presentation of COVID-19 is frequently asymptomatic in 85% of the cases, though 15% of cases are presented with moderate to severe forms of the disease owing to the progress of acute lung injury (ALI). Also, 5% of COVID-19 patients may be seriously affected and need ventilation due to the progression of acute respiratory distress syndrome (ARDS) (Al-Kuraishy et al. 2021).

ACE2 is a peptidase that metabolizes the vasoconstrictor angiotensin II (Ang II) to the vasodilator Ang1-7 and Ang1-9. Thus, reduction of ACE2 during SARS-CoV-2 infection persuades vasoconstriction and development of endothelial dysfunction (ED), oxidative stress (OS), and inflammatory disorders (Al-Kuraishy et al. 2021). These changes cause hypoxia and cytokine storm with the development of cardio-pulmonary complications (Al-Kuraishy et al. 2021). In reaction to diverse viral infections, pro-inflammatory, anti-inflammatory cytokines, and bioactive lipids are released from the immune cells (Onohuean et al. 2021). Bioactive lipids and other polyunsaturated fatty acids participate in a vital role in the process of viral infections including SARS-CoV-2 infection (Das 2020). One of these mediators is the anti-inflammatory lipoxin (LX).

As LXs are potent anti-inflammatory mediators, we aimed at reviewing the potential role of LXs in COVID-19 regarding the effects of LXs on the inflammatory signaling pathways which are linked with COVID-19 pathogenesis.

### Characteristic of lipoxin

**Fig. 1:**
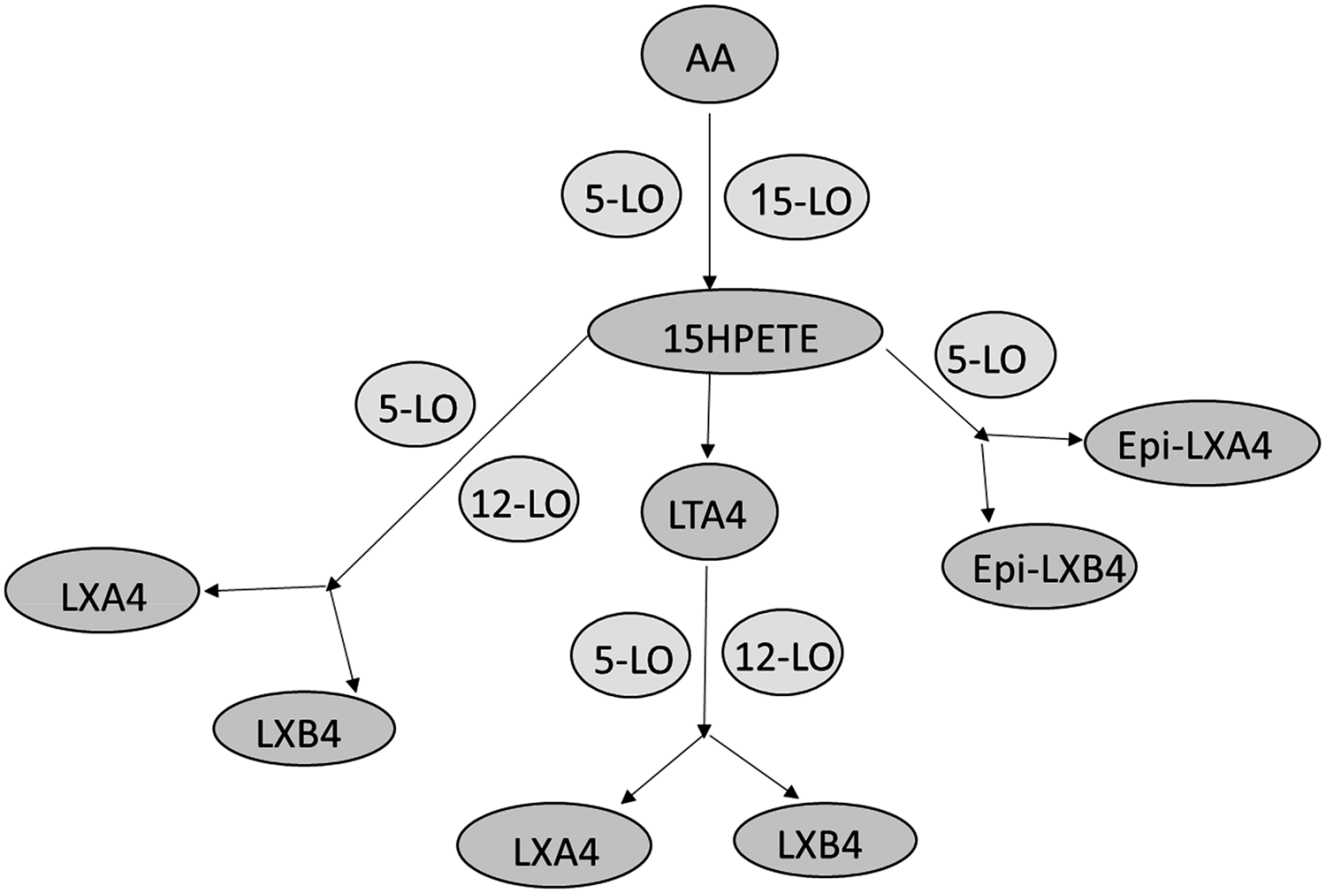
Biosynthesis and pathway of lipoxins (LXs) from arachidonic acid (AA) through lipoxygenase (LO) 5 and 15. AA is converted to 15-hydroxy peroxy eicosatetraenoic acid (15HPETE), which form LTA4 that directly forms LXA4 and LXB5. The 15HPETE can form LXA4 and LXB5 through 5/12-LO, or can form Epi-LXB4 and Epi-LXA4 through 5-LO

LX is a bioactive autocoid from arachidonic acid (AA) made by different cell types. Two types of LX are identified, LXA4 and LXB4, in addition, two epimers are recognized and they are 15-epi-LXA4 and 15-epi-LXB4 (Bäck et al. 2014). LXs were first described in 1984 by Laurate Samuelsson and his colleagues (Serhan et al. 1984). Succeeding studies confirmed that LXs had anti-inflammatory effects by resolving inflammation in different infectious and non-infectious inflammatory disorders (Hughes et al. 2017). LXs and epimers inhibit chemotaxis of neutrophils, macrophage activations, the release of pro-inflammatory cytokines, and antibody productions from B cells. As well, LXs block various ligands involved in the release of pro-inflammatory cytokines such as formyl peptide receptor (FPR) (Bai et al. 2019). LXs are generated by collaboration between 5-lipoxygenase (5-LO) and 12–15 LO during cell interactions (Fig. 1). The 15-epi-LXs are formed and designated by aspirin acetylated cyclooxygenase 2 (COX-2) in collaboration with 5-LO. The 15-epi-LXA4 is known as aspirin-triggered LX (ATL). LXA4 receptors were initially named FPR, which was renamed ALX receptors (Romano et al. 2007).

LXs are generated from AA by 5-LO via transcellular and unicellular biosynthetic pathways. Specifically, in the trans-cellular pathway, LXs are formed by 12-LO derived from platelet-neutrophil interaction, while in the unicellular pathway LXs are formed by 5-LO (Prieto et al. 2010). In addition, two different pathways for biosynthesis of LXs are triggered that are aspirin-triggered (AT) and statins triggered (ST) LXs. Aspirin induces acetylation of COX-2 that increases AA substrate for 5-LO, while statins increase conversion of AA to 15-LXs (Petri et al. 2017; Planagumà et al. 2010).

LXs are metabolized by macrophages 15-hydroxyprostaglandin dehydrogenase to inactive metabolites. However, 15-epi-LXA4, 15-epi-LXB4, and LX synthetic analogs are resistant to being metabolized. LXs enhance brain endocannabinoids and exert similar effects to the endogenous endocannabinoid system. LXA4 exerts a protective effect against memory impairment in animals through the activation of cannabinoid receptor 1 (CB1) (Pertwee 2012).

Moreover, specialized pro-resolving lipid mediators (SPMs) which are formed from polyunsaturated fatty acids (PUFAs) during acute and chronic inflammations, are involved in the resolution process (Bannenberg and Serhan 2010). The SPMs play a role in the reduction of neutrophil infiltration, induction of neutrophil apoptosis and/or efferocytosis, and counter-regulation of cytokines and chemokines with induction of macrophage polarization toward anti-inflammatory M2 type (Bannenberg and Serhan 2010).

Taken together, the main functions of LXs are inhibition of pro-inflammatory cytokines, activation of anti-inflammatory cytokines, activation of phagocytosis by monocytes/macrophages, and removing apoptotic neutrophils (efferocytosis), and suppression of the release of reactive oxygen species (ROS). This is in addition to increasing the production of the anti-platelet prostaglandin (prostacyclin I2) with a vasodilator effect by releasing nitric oxide (NO) (Luo et al. 2013; Miao et al. 2015). Besides, LXs promote the release of anti-inflammatory cytokines by increasing the entry of nuclear factor erythroid 2 related factors (Nrf2) which increases the production of heme oxygenase 1 (HMOX-1) (Wu et al. 2013). Further, HMOX-1 improves the synthesis of antioxidant glutathione which neutralizes ROS/peroxynitrite and prevents the oxidative stress (OS) (Wu et al. 2013).

It has been reported that LXB4 and other analogs attenuate inflammatory signaling pathways such as nuclear factor kappa B (NF-κB) and activated protein 1 (AP-1) (Liu et al. 2018). As well, LXB4 stimulates the expression of the suppressors of the cytokine signaling (SOCS) proteins. This inhibits the activation of the signal transducer and activator transcription (STAT), leading to an increase in the expression of the pro-inflammatory cytokines (Machado et al. 2006). Indeed, LXA4 can bind aryl hydrocarbon receptor (AHR) and activate nuclear xenobiotic response elements causing activation of the expression of the anti-inflammatory cytokines (Abma et al. 2020). LXs are effective against various inflammatory disorders including bacterial sepsis-induced ALI/ARDS, respiratory syncytial virus (RSV) induced-ALI, and different parasitic infections (Lee et al. 2017). As well, LXs reduce allergic reactions and inflammation in asthma by reducing leukotriene C (LTC) (Liu et al. 2021).

**Fig. 2:**
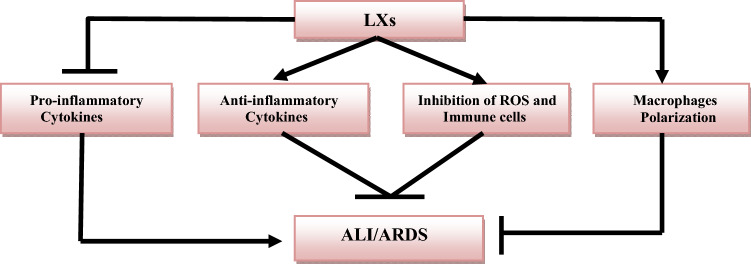
Role of lipoxins (LXs) in the prevention of acute lung injury (ALI) and acute respiratory distress syndrome (ARDS)

Taken together, LXs have anti-inflammatory and antioxidant effects by reducing the release of the pro-inflammatory cytokines and the generation of ROS. Therefore, LXs or LX receptor agonists could be effective against different inflammatory disorders (Fig. 2).

### Role of lipoxin in respiratory disorders

It has been reported that LXA4 can reduce pulmonary inflammation and bronchial hyper-responsiveness, so LXA4 agonists and analogs could be a new therapeutic strategy in the management of asthma (Kong et al. 2017). A pilot study involved 50 asthmatic patients treated with LXA4 methyl ester compared to inhaled bronchodilators showed that LXA4 methyl ester was more effective with fewer adverse effects compared to the standard anti-asthmatic therapy (Kong et al. 2017). Ricklefs et al. found that expression of ALX receptors is correlated with lung inflammation and asthma severity (Ricklefs et al. 2017). Besides, a case–control study comprised 22 asthmatic children compared with 22 healthy persons found that LXA4 serum level is correlated with asthmatic severity (Mohamed et al. 2020). Furthermore, LXs which reduce neutrophil recruitments are decreased in patients with cystic fibrosis compared to the controls (Karp et al. 2004). Administration of LXs analogs inhibits neutrophil-mediated inflammation, and decreases bacterial inflammation and the severity of cystic fibrosis (Karp et al. 2004). Thornton et al. illustrated that LXA4 promotes antibiotic efficacy against *Pseudomonas aeruginosa* by reducing the expression of virulence genes in patients with cystic fibrosis (Thornton et al. 2021).

LXs are also effective against the development of ALI and ARDS, an experimental study showed that mesenchymal cells attenuate the development of ALI in mice by activating the release of LXA4 (Fang et al. 2015). Repair of the lung alveolar structures and resolution of ALI required a balance between inflammatory molecular signals and inflammatory reactions that need an active metabolic and biochemical process (Fang et al. 2015). LXs have dual pro-resolution and anti-inflammatory activities, thereby reducing the pulmonary epithelial and endothelial permeability (Fang et al. 2015; Levy et al. 2001). LXs through interaction with ALX/FPR2 promote the alveolar fluid clearance and resolution of the inflammations in ALI (Fang et al. 2015). El-Kebir et al. revealed that 15-Epi-LXA4 enhances the resolution of ALI by inhibiting myeloperoxidase (MPO)-induced stimulation of extracellular signal-regulated kinase-mediated phosphorylation (Kebir et al. 2009). MPO is released from neutrophils and acts as a paracrine and autocrine mediator increasing MPO release and delaying the intrinsic apoptosis of neutrophils (Mayadas and Cullere 2005).

LXA4 through its anti-inflammatory action inhibits the expression of neutrophils Mac-1 and other adhesion molecules (Chiang et al. 2006). Ye et al. observed that LXA4 inhibits acute pancreatitis-induced ALI through modulation of Nrf2/HO-1 and suppression of generation of ROS-induced inflammation and development of ALI (Ye et al. 2019). Moreover, Wang et al. review study found that various anti-inflammatory mediators including LXA4 play a critical role in the enhancement of the alveolar fluid clearance in ARDS (Wang et al. 2018). A systematic review regarding the role of the inflammatory mediators in the progression of ARDS revealed that aspirin through inhibition of COX-2 increases the production of ATL, which inhibits NF-κB and production of IL-8. ATL promotes phagocytosis of apoptotic neutrophils and reduces the severity of ARDS (Toner et al. 2015).

Concerning the role of LXs in respiratory viral infections, Ciloniz et al. an experimental study observed that dysregulation of the anti-inflammatory LXA4 is associated with lethal H5N1 influenza infection in mice due to the reduction of the protective effect of LXA4 (Cilloniz et al. 2010). As well, H5N1 influenza infection induces a reduction in the expression of SOCS2, which is needed in the action of LXA4 (Russell and Schwarze 2014). Therefore, the administration of LXA4 analogs may reduce the severity of H5N1 infection. It has been shown that respiratory syncytial virus (RSV) infection in 5-LO deficient mice is exaggerated causing severe pulmonary inflammation compared with wild-type mice (Shirey et al. 2014). Treatment with LXA4 analogs in 5-LO deficient mice with RSV infection restored the body's anti-inflammatory capacity against respiratory viral infections (Shirey et al. 2014). Moreover, alveolar macrophages subjected to different viral infections produce a large amount of LXA4 (Kim 1990). Thus, a higher LXA4 concentration is linked with various respiratory viral infections and could be a surrogate biomarker in this regard. Therefore, LXA4 receptor antagonists may reduce the resolution of viral and bacterial-induced ALI (Gotts et al. 2018).

**Fig. 3:**
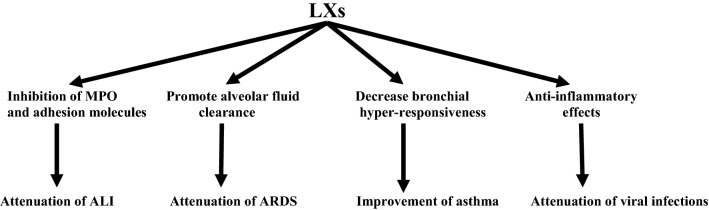
Protective effects of lipoxins (LXs) in respiratory disorders: LXs attenuate acute lung injury (ALI) through inhibition of neutrophil myeloperoxidase (MPO). They also attenuate acute respiratory distress syndrome (ARDS) by improving the alveolar fluid clearance and improve asthma by reducing bronchial hyper-responsiveness. Moreover, they attenuate respiratory viral infections by their anti-inflammatory effects

These observations support the potential role of LXs in attenuating respiratory viral infections through the modulation of anti-inflammatory response. Thus, LXA4 receptor agonists or LXA4 analogs could be novel therapeutic modalities in attenuating respiratory viral infections and their associated complications like ALI and ARDS (Fig. 3).

### Role of lipoxin in SARS-CoV-2 infection

Bioactive lipids including the pro-inflammatory mediators like prostaglandins (PGs), leukotrienes (LTs), and thromboxane A2 (TXA2), and the anti-inflammatory mediators like LXs, protectin, maresins, and resolvins are formed from AA during acute inflammations and infections. These bioactive lipid mediators augment macrophage phagocytic activity and at the same time resolve the inflammatory process, and enhance microbial clearance (Norris et al. 2018). It has been shown that the bioactive lipid, LXs, and AA, can modulate SARS-CoV-2 infection through inhibition of viral entry, inhibition of viral replication, downregulation of the expression of ACE2, and suppression of the pro-inflammatory cytokines (Das 2021). Therefore, deficiency of AA and bioactive lipid mediators may augment viral infections due to interruption of the anti-inflammatory process. Pal et al. reported that the paucity of SPMs in obese patients increases the risk of SARS-CoV-2 infection (Pal et al. 2020). Thus, oral or intravenous administrations of AA and LXs could be effective in COVID-19 by increasing the resistance and recovery from SARS-CoV-2 infection (Das 2021). Lee observed that SPMs mainly LXs are regarded as a potential therapy against SARS-CoV-2 infection through modulation of the viral-inflammation circuits (Lee 2021). Regidor et al. proposed that SPMs can reduce the severity of SARS-CoV-2 infection and its associated complications including ALI, immunothrombosis, and cytokine storm via attenuating the release of the pro-inflammatory cytokines (Regidor et al. 2020). Likewise, Hammock hypothesized that SARS-CoV-2 infection is linked with endoplasmic stress and the development of eicosanoids storm due to the generation of pro-inflammatory cytokines (Hammock 2020). Therefore, anti-inflammatory drugs, via increasing the anti-inflammatory LXs, may counteract eicosanoids storm in COVID-19.

LXs and LXs receptor agonist, BML-111, can induce autophagy in lung alveolar macrophages and protect from ALI through modulation of the mitogen-activated protein kinase (MAPK) signaling pathway (Liu et al. 2018). Besides, different studies revealed a potential role of LXs in preventing ALI and ARDS (Fang et al. 2015; Levy et al. 2001; Kebir et al. 2009; Mayadas and Cullere 2005). So, LXs and LXs receptor agonists might be effective in the reduction of SARS-CoV-2 infection-induced ALI/ARDS by modulation of the hyperinflammation and inflammatory signaling pathways.

Of note, SARS-CoV-2 infection is associated with activation of various inflammatory signaling molecules including NF-κB, STAT3, MAPK, mammalian target of rapamycin (mTOR), and nod-like receptor pyrin 3 (NLPR3) inflammasome. Activation of these inflammatory molecules is linked with the development of hyperinflammation, cytokine storm, ED, OS, immunothrombosis, pulmonary thromboembolic disorders, and ALI/ARDS (Rex et al. 2021; Shah et al. 2020). Inhibition of these inflammatory signaling molecules may reduce COVID-19 severity and the associated complications. Interestingly, LXs inhibit NF-κB, AP-1, and STAT3 in different experimental studies and clinical trials (Liu et al. 2018; Abma et al. 2020). Besides, LXs block the activation of human MAPK, (Chen et al. 2014) and they can reduce inflammation by inhibiting mTOR in Kaposi sarcoma-associated herpes viral infection (Chandrasekharan and Sharma-Walia 2015). Furthermore, they can suppress the activation of NLPR3 inflammasome in chronic obstructive pulmonary disease (Cao et al. 2018). Thus, LXs may reduce the inflammatory changes induced by activation of MAPK, mTOR, and NLPR3 inflammasome in COVID-19.

In addition, SARS-CoV-2 induces a paradoxical activation of SOCS and abnormal immune response (Johnson et al. 2020). Johnson and his colleagues suggested that SOCS antagonists might be beneficial in this regard. However, using of these antagonists led to severe exacerbation of COVID-19 due to the uncontrolled release of pro-inflammatory cytokines (Johnson et al. 2020). Of note, LXB4 stimulates SOCS signaling with subsequent inhibition of STAT3-dependent release of the pro-inflammatory cytokines (Machado et al. 2006). Therefore, LXs is regarded as an endogenous inhibitor that prevents the progression of inflammatory disorders and may reduce SARS-CoV-2-induced hyperinflammation and complications.

In COVID-19, Nrf2 which inhibits the production of ROS and induces the release of the anti-inflammatory cytokines is inhibited by SARS-CoV-2 leading to OS and inflammatory disorders (Cuadrado et al. 2020). For this reason, activation of Nrf2 can decrease the inflammation, OS, and restore tissue repair in both ALI and ARDS (Cuadrado et al. 2020). Besides, SARS-CoV-2 infection, old age, and metabolic disorders are associated with low levels of stress protein in particular HMOX-1 protein. Although, exaggerated immune response and hyperinflammation induce the expression of HMOX-1 to counterbalance the inflammatory burden (Hooper 2020). Activators of HMOX-1 such as melatonin, resveratrol, statins, and curcumin can improve COVID-19 outcomes (Hooper 2020). An observational cohort study illustrated that HMOX-1 serum level was increased in patients with severe COVID-19 and correlated with low oxygen saturation (Hooper 2020). Herein, LXs, which encourage the release of the anti-inflammatory cytokines via increasing the entry of Nrf2 and inducing HMOX-1, (Wu et al. 2013) could be beneficial in the mitigation of hyperinflammation and OS in COVID-19.

Furthermore, AHR which is activated by LXs has a protective effect against lung hyper-responsiveness and injury through modulation of the cell recruitments and inflammation (Michaudel et al. 2020). Bock revealed that AHR has anti-inflammatory and pro-inflammatory roles by releasing LXs and pro-inflammatory cytokines, respectively (Bock 2020). AHR is mainly involved in the resolution of the inflammatory process through the LXs-dependent pathway (Bock 2020). However, Anderson et al., proposed that AHR is implicated in the pathogenesis of SARS-CoV-2 infection and it is linked with COVID-19 severity (Anderson et al. 2020). Therefore, AHR antagonists could be prophylactic and therapeutic agents in COVID-19 severity. This observation raises a clue about the controversy concerning the relation of AHR with LXs in COVID-19.

Indeed, LXs have a potent anti-inflammatory effect through inhibition of the expression of toll-like receptor 4 (TLR-4) and myeloid differentiation gene 88 (MyD88), which are involved in NF-κB activation and release of the pro-inflammatory cytokines (Ali et al. 2020). In COVID-19, the TLR-4/MyD88 axis is activated and associated with the release of pro-inflammatory cytokines and the development of cytokine storm (Cuevas et al. 2021). Thus, LXs through inhibition of TLR-4/MyD88, may reduce SARS-CoV-2-induced hyperinflammation and cytokine storm in COVID-19.

Also, immunothrombosis and pulmonary thromboembolic disorders are common hallmarks of COVID-19 due to ED, OS, and hyperinflammation (Loo et al. 2021). Yeung et al. illustrated that 15-LO metabolites, mainly LXs, have anti-platelet effects thereby reducing clot formation and maintaining normal body homeostasis (Yeung and Holinstat 2011). Similarly, LXs activate the release of tissue factors which inhibit the induction of clotting factors and the development of thrombosis (Maderna et al. 2000). With these findings, LXs and LXs analogs and/or receptor agonists may mitigate thrombotic events in COVID-19 and reduce the risk of pulmonary micro-thrombosis.

Remarkably, leukotrienes (LTs) are triggered during SARS-CoV-2 infection leading to pulmonary and extra-pulmonary inflammatory changes with the risk of development of cytokine storm (Al-Kuraishy et al. 2021). Al-kuraishy et al. found that LT antagonists can alleviate pulmonary and extra-pulmonary manifestations of COVID-19 (Al-Kuraishy et al. 2021). It has been reported that LXs reduce the expression of LTs and attenuate the associated inflammatory disorders (Papayianni et al. 1996). In this sense, LXs and LXs analogs can mitigate COVID-19 manifestations through suppression of the LT pathway.

Additionally, SARS-CoV-2 infection induces down-regulation of ACE2 with subsequent elevation of vasoconstrictor and pro-inflammatory AngII with reduction of the vasodilator and protective Ang1-7 and Ang1-9 (Elekhnawy and Negm 2022). High circulating AngII in COVID-19 patients triggers cardio-pulmonary complications including pulmonary thrombosis and ALI/ARDS (Elekhnawy and Negm 2022). Recently, it has been shown that LXA4 inhibits the excitatory auto-antibodies against the angiotensin 1 receptor (AT1R) of AngII and prevents the development of preeclampsia (Liu et al. 2020). Chen et al. study revealed that LX agonist BML-111 protects and prevents the occurrence of ALI and hepatic injury by reduction of ACE activity and increasing the activity of ACE2 and Ang1-7 (Chen et al. 2019). Therefore, LXs and LXs agonists through regulation of the renin-angiotensin system (RAS) can attenuate AngII-induced cardiopulmonary complications in COVID-19.

Moreover, expression of the anti-inflammatory FPRs is reduced in COVID-19 due to an unbalanced immune-inflammatory response leading to a delay in the resolution of inflammatory changes (Perretti and Godson 2020). FPRs are activated by LXs leading to anti-inflammatory effects (Ge et al. 2020). Therefore, LXs/FPRs axis may reduce SARS-CoV-2 infection-induced hyperinflammation. Besides, the anti-inflammatory effects of LXs are mediated by increasing the release of NO through activation of nitric oxide synthase (NOs) (Paul-Clark et al. 2004). In SARS-CoV-2 infection, there is severe ED and a reduction in the release of NO (Green 2020). Restoration of NO by dietary nitrate improves the endothelial function and prevents platelet-endothelial interaction and the development of thrombosis (Green 2020). In this sense, LXs analogs could be potential agents in the attenuation of ED caused by NO deficiency.

Without a doubt, LXs increase the polarization of macrophages from classical inflammatory (M1) to the alternative anti-inflammatory (M2), thereby reducing the inflammatory effect of macrophages (Li et al. 2021). In SARS-CoV-2 infection, M1 macrophages are predominant leading to an increase in the release of the pro-inflammatory cytokines and hyperinflammation (Ferraro et al. 2021).

Finally, LXs are regarded as endogenous activators of the CB1 receptor; promoting the activity of the endocannabinoid system (ECS) (Pertwee 2012). It has been shown that ECS is involved in the regulation of the immune response by inhibiting the release of the pro-inflammatory cytokines and improving the release of the anti-inflammatory cytokines (Briand-Mésange et al. 2020). In COVID-19, activation of ECS is associated with a reduction in the severity of SARS-CoV-2 infection through inhibition of the development of cytokine storm (Onaivi and Sharma 2020).

Taken together, LXs have immunomodulating effects on SARS-CoV-2 infection in COVID-19 through modulation of the immune cells and response of monocytes/macrophages phagocytic activity with inhibition of inflammatory signaling pathways. Therefore, LXs and LXs analogs could be effective in the management of COVID-19.

**Fig. 4:**
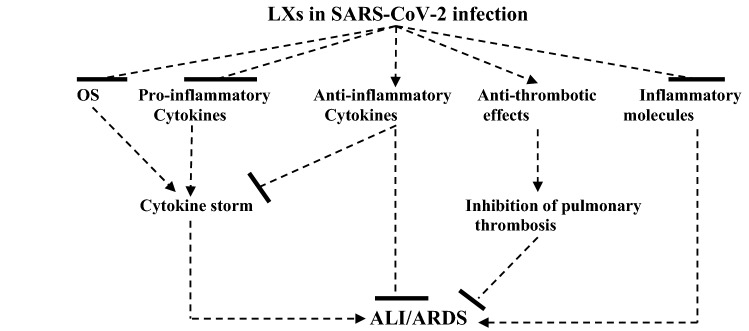
Role of lipoxin (LXs) in SARS-CoV-2 infection: LXs inhibit oxidative stress (OS), pro-inflammatory cytokines, and inflammatory molecules with anti-inflammatory and anti-thrombotic effects. These effects inhibit the development of cytokine storm and pulmonary thrombosis with subsequent suppression of the development of ALI and ARDS

The present study had several limitations including a paucity of preclinical trials and prospective clinical studies regarding the role of LXs in COVID-19. However, this narrative review shed light on the anti-inflammatory role of LXs in COVID-19 and gave suggested mechanisms for the beneficial effect of LXs in COVID-19 (Fig. 4).

## Conclusions

LXs have anti-inflammatory effects by inhibiting the inflammatory signaling pathways and releasing the pro-inflammatory cytokines with augmentation of the anti-inflammatory cytokines. Taken together, LXs, LXs analogs, and LXs agonists could be effective therapeutic modalities in the management of COVID-19. These observations need to be confirmed by experimental, preclinical, and clinical studies.

## Data Availability

All data are available in the manuscript.
